# Is the Precipitation of Anxiety Symptoms Associated with Bolus Doses of Flumazenil a Barrier to Its Use at Low Continuous Doses in Benzodiazepine Withdrawal?

**DOI:** 10.3390/jcm11195948

**Published:** 2022-10-08

**Authors:** Alexander Gallo, Tim MacDonald, Kellie Bennett, Gioiamia Basso-Hulse, Gary Hulse

**Affiliations:** 1 Division of Psychiatry, Medical School, The University of Western Australia, Nedlands 6009, Australia; 2 Fresh Start Recovery Programme, Subiaco 6008, Australia; 3 Currumbin Clinic, Currumbin 4223, Australia; 4 School of Medicine, Griffith University, Gold Coast 4215, Australia; 5 School of Medical and Health Sciences, Edith Cowan University, Joondalup 6027, Australia

**Keywords:** flumazenil, infusion, benzodiazepine, anxiety, GABA

## Abstract

Introduction: Benzodiazepines (BZDs) are used in the management of anxiety and sleep disorders; however, chronic use is associated with tolerance and dependence. During withdrawal, symptoms of anxiety are often severe and problematic for patients and may lead to relapse or maintenance on low doses of BZDs. Low, continuous doses of flumazenil reduce BZD withdrawal symptoms in several studies; however, bolus doses are known to induce anxiety and precipitate panic. Accordingly, this study aimed to determine whether continuous low-dose flumazenil is anxiogenic like bolus doses. Method: In a randomised control cross over design, participants received a continuous low-dose flumazenil infusion for eight days at an approximate rate of 4 mg/24 h or placebo before crossing over to the alternate study arm. Participants were able to request diazepam as needed. The primary outcome was the change in state anxiety levels. Trait anxiety was also recorded at baseline and one month after the flumazenil/placebo infusion period. Results: BZD use was significantly reduced in both groups. There were no significant differences between state anxiety and the 95% confidence interval showed no evidence of a clinically significant anxiogenic effect from low-dose flumazenil. Trait anxiety was significantly reduced one month after the infusion period. Conclusion: There is no evidence that continuous low-dose flumazenil infusion significantly increases state anxiety levels to a clinically significant level. Interestingly, flumazenil may decrease state anxiety during BZD withdrawal, unlike bolus doses of flumazenil. Flumazenil may have an anxiolytic effect on trait anxiety, which was evident one month after treatment.

## 1. Introduction

Benzodiazepines (BZD) are a class of anxiolytics acting on the BZD or gamma aminobutyric acid A (GABA_A_) receptor. It is widely accepted that chronic exposure to therapeutic and higher doses of BZDs leads to tolerance and dependence [1,2,3,4,5] via a number of a neuroadaptive mechanisms [6,7,8,9]. Tolerance to the sedative effects of BZDs typically occurs after two weeks of continued use [10], while tolerance to the anxiolytic effects rarely occurs [6]. Withdrawal syndromes are associated with both low and high doses of BZDs, with symptoms including rebound anxiety, sleep disturbances, panic attacks, irritability, and in severe cases, seizures [11,12]. While successful withdrawal is contingent on the management of all symptoms, arguably, the most pertinent symptom of withdrawal is rebound anxiety, which can lead to relapse to BZD use [13,14]. Additionally, BZDs are not uncommonly prescribed as part of the management of anxiety disorders [15]; therefore, withdrawal symptoms may be compounded by the re-emergence of the anxiety disorder initially treated. Currently, withdrawal from BZDs is generally managed by slowly tapering the dose over weeks to months, even years; however, this method is still associated with increased anxiety and withdrawal symptoms [16].

A number of pharmacological options to aid BZD withdrawal have been investigated [5]. Flumazenil, a competitive antagonist at the allosteric benzodiazepine binding site with some evidence for a weak agonistic action [17], is one agent with a small but increasing body of evidence for its use in BZD withdrawal. For this indication, it is employed in low doses (i.e., 1–4 mg), and delivered intravenously or subcutaneously over extended periods (e.g., 24 h). Conversely, some evidence suggests that flumazenil, when delivered as bolus intravenous doses of 1–3 mgs, precipitates withdrawal, even in chronic low-dose BZD users (mean diazepam equivalent of 11.2 mg/day), anxiety [18,19,20,21,22,23] and panic attacks [24]. Low doses of 1–3 mgs of flumazenil precipitating these anxiety symptoms were delivered intravenously over seconds to minutes, which contrasts to similar or higher doses used in BZD withdrawal where flumazenil delivery has been over several hours.

While flumazenil doses in BZD withdrawal typically range from 1–4 mg/24 h (see Gallo and Hulse 2021 for review [25]), there is no explicit anxiety data from a randomised control setting to suggest whether an anxiogenic response is observed. Accordingly, the aim of this study was to explore the extent to which continuous low-dose flumazenil (4 mg/24 h), delivered over eight days, was anxiogenic or anxiolytic during BZD withdrawal measured by the Spielberger State Trait Anxiety Inventory–State (STAI–S). The minimum clinically important difference (MCID) for the state anxiety inventory was set at 10 points as described in previous studies [26,27].

## 2. Method

### 2.1. Participants

Participants were part of a multi-site double-blind randomised control crossover trial for BZD withdrawal using subcutaneous low-dose flumazenil infusion, which has been previously reported [28]. Inclusion criteria were (1) daily BZD use for more than three months, (2) daily BZD use of at least 10 mg diazepam equivalents (except for one participant taking 1 mg lorazepam, which has a range of 5–10 mg diazepam equivalent) [29], and (3) a desire to stop BZDs. Exclusion criteria were (1) history of epilepsy or seizure/fit due to the risk of seizures with low-dose flumazenil treatment [30], (2) currently pregnant or breastfeeding, and (3) under the age of 18. Participants were treated as both inpatients and outpatients and were under the care of physicians in Currumbin, Queensland and Subiaco, Western Australia.

### 2.2. Study Design

This study forms part of a double-blind randomised control trial (ACTRN12616001560482; anzctr.org.au). Participants were randomly assigned to receive flumazenil or placebo infusions before crossing over to the alternative arm of the study with both staff and patients blinded to treatment allocation. During this time, participants could request up to 10 mg diazepam on an as needed basis with no daily maximum dose. This design was used to ensure all participants received flumazenil treatment, as many of the participants had previously failed to withdraw. As such, the focus of this study was to assess anxiety outcomes before the cross-over period during continuous low-dose flumazenil infusion compared to placebo. Data were collected between 2017 and 2019.

### 2.3. Procedure

Baseline BZD use was calculated during participant screening. Total BZD dose was converted to a daily diazepam equivalent using the South Australia Benzodiazepine Equivalents Table [29]. The conversion guide considers 5 mg diazepam to be equivalent to 0.5 mg alprazolam, 0.25 mg clonazepam, 1 mg lorazepam, 30 mg oxazepam, 10 mg temazepam, 10 mg zolpidem, and 7.5 mg zopiclone.

Following baseline assessment and randomisation, participants received treatment in one of the two groups: (group 1) two flumazenil (16 mg/30 mL, each delivered in approximately 96 h for a total of 32 mg over approximately eight days) infusions followed by two placebo infusions of the same volume and duration (FP), or (group 2) two placebo infusions followed by two flumazenil infusions (PF). Flumazenil/placebo were administered consecutively and subcutaneously using the SpringFusor^®^ pump manufactured by Go Medical Industries (Subiaco, Western Australia). The SpringFusor^®^ pump is designed to deliver a 30 mL dose continuously over approximately 96 h, delivering 4 mg/24 h ± 20%, which has been previously described [31]. Participants were able to request up to 10 mg diazepam if they scored a total of two or more on the modified six-item Clinical Institute Withdrawal Assessment Scale–Benzodiazepines (CIWA–B) [32], which assessed difficulty concentrating, racing heartbeat, loss of appetite, anxiety, headaches, and observable behaviour. Clinical staff were trained by the treating physician on the scoring of the modified CIWA–B to administer diazepam. There was no maximum daily diazepam dose. Participants were instructed to cease all BZDs outside of the trial protocol and were maintained on other regular medications. Any BZD use outside of the protocol was recorded and included in the daily BZD use. Participants not already receiving anti-epileptic medications were commenced on phenytoin 100 mg three times per day for the duration of the flumazenil/placebo infusions due to the risk of seizures associated with BZD withdrawal and flumazenil treatment [30]. Phenytoin was ceased at the conclusion of the placebo/flumazenil infusions. The method has been previously described [28].

### 2.4. Outcomes

The primary outcome was the change in state anxiety levels from baseline. BZD use was recorded daily on a BZD record sheet and has previously been reported for this cohort but was separated into four groups based on their BZD use at baseline [28]. To explain changes in state anxiety levels, BZD data was needed as an aggregate for group 1 (FP) and group 2 (PF), without being further separated into participants taking high (≥30 mg diazepam equivalent at baseline) and low doses (<30 mg diazepam equivalent at baseline). BZD recording started on the day of the infusion start; however, due to differences in the time of infusion insertion and removal, day one in the analysis represents BZD use from 08:00 the day after the infusion was inserted and does not include the day the infusion was removed. Accordingly, there are only six days of BZD use recorded for each eight-day infusion period. Anxiety levels were measured using the Spielberger State-Trait Anxiety Inventory [33]. State anxiety was measured at baseline, day 4, 8, 9, 12, and 16 during the infusions. Trait anxiety was measured at baseline and 30 days post-infusion. Comparisons were not made for data on day 9, 12, and 16 as there is a theoretical basis behind an anxiolytic action of flumazenil in anxiety disorders [34]; as such, using group 1 (FP) as a comparison after being treated with flumazenil may reveal larger differences in anxiety levels between groups, which are not attributed to an anxiogenic response to flumazenil in group 2 (PF).

### 2.5. Randomisation and Blinding

Randomisation codes were computer generated with a block size of two and a 1:1 allocation ratio. A unique identification number was printed on the label of each vial with a corresponding key provided in a sealed envelope. Randomisation was managed by the research officer at the Subiaco site. The research officer had no other involvement in the trial. Once the participant was recruited, the research officer could instruct which flumazenil/placebo vials should be used. Flumazenil/placebo vials were labelled “flumazenil/placebo 16 mg” and numbered one to four for the order of treatment. All other clinical and research staff were blinded to participant allocation throughout the duration of the trial.

### 2.6. Statistical Methods

Mean diazepam use on each day, average diazepam use over the six-day diazepam recording period, and mean anxiety score across day one, four, and eight were calculated followed by the difference between baseline and average anxiety and baseline and day one anxiety for the first infusions before the crossover period. Comparisons between groups were performed using an independent samples *t* test if assumptions of normality and homogeneity of variance were met. Where a significant Levene’s test indicated a violation of homogeneity of variance, Welch’s *t* test was used. Where the assumption of normality was violated, a Mann–Whitney *U* test was used. A paired samples *t* test was used to measure any significant differences in daily BZD use at baseline and the average over the duration of the first infusions before the crossover period. If assumptions of normality were violated, a Wilcoxon Signed Rank test was used. A paired samples *t* test was used to compare trait anxiety levels in the entire cohort from baseline to 30 days post the end of the last infusion (flumazenil or placebo) if normality of group scores and differences was demonstrated. If assumptions of normality were violated, a Wilcoxon Signed Rank test was used. Non-significant differences were examined by estimating a Bayes factor.

### 2.7. Missing Data

Number of missing items on the STAI-S out of 26 participants at baseline, day one, four, and eight follow-ups ranged from 0 (0%) to 2 (7.7%). The maximum number of items missing for any one participant was four (20%). All data were missing for one participant in group 1 (FP) on day 1; however, mean anxiety results were still calculated as the mean of day four and eight for the average anxiety analysis. Missing scale scores were present for the Spielberger State Trait Anxiety Inventory–Trait (STAI–T) for five participants and, as such, there were only data for 21 participants. Number of missing items on the STAI–T ranged from 0 (0%) to 1 (4.8%). The maximum number of items missing for any one participant was six (30%). Accordingly, missing items were imputed for both scales using a simple mean imputation within each patient, which involves using the mean score of the variable items’ responses within a participant to replace the missing item response(s). This method is reasonable since the items in psychometric scales are correlated with each other and is appropriate when more than 50% of items have a valid response [35]. Missing items that are scored inversely were adjusted accordingly. Diazepam use data were missing for one participant.

### 2.8. Ethics and Consent

The study was reviewed and approved by Southcity Medical Centre Human Research Ethics Committee (002/2015) with recognition by The University of Western Australia Human Research and Ethics Committee (RA/4/1/7970) and approved by Griffith University Human Research Ethics Committee (2016/559). All participants gave written informed consent prior to enrolment into the trial.

## 3. Results

### 3.1. Baseline Participant Characteristics

Participants were mostly female in group 1 (FP) (61.5%) and group 2 (PF) (69.2%). Daily diazepam equivalent use at baseline was higher in group 1 (FP) (50.5 mg) compared to group 2 (PF) (33.0 mg); however, these differences are not statistically significant and largely due to one participant taking 280 mg daily diazepam equivalents at baseline in group 1 (FP). Diazepam equivalents at baseline in group 1 (FP) ranged from 10–280 mg and 5–100 mg in group 2 (PF). Clonazepam was the most used BZD at baseline and all participants had a diagnosed psychiatric condition. The most common psychiatric diagnosis was depression followed by an anxiety disorder (Table 1).

### 3.2. Diazepam Use across Flumazenil and Placebo Infusions

Diazepam use decreased from baseline over the first eight-day infusion period in both groups (Table 2). A Wilcoxon Signed Rank test showed that mean diazepam use from baseline to mean use over the first eight-day infusion period was significantly lower in group 1 (FP), *T* = 9.0, *z* = −2.35, *n* = 12, *p* = 0.019, *r* = −0.68 and group 2 (PF), *T* = 4.5, *z* = −2.87, *n* = 13, *p* = 0.004, *r* = −0.80. Of importance, both groups had approximately the same mean daily BZD dose over the first eight days of the trial (Table 2). The BZD use results have previously been reported, which showed a significant reduction in BZD use in group 1 (FP) compared to group 2 (PF); however, this was only seen in high dose users (≥30 mg diazepam daily equivalent dose) [28].

### 3.3. State Anxiety Levels between Groups during Infusions

An independent samples *t* test was used to compare the average difference in anxiety levels between baseline and average anxiety over the first eight-day flumazenil infusion period for group 1 (FP) (*n* = 13) and placebo infusion for group 2 (PF) (*n* = 13). Group 1 (FP) showed a mean reduction in state anxiety score of 7.37 while group 2 (PF) showed a reduction of 0.25. The mean difference in anxiety between groups was not statistically significant, *M* = −7.12, [95% CI −16.14, 1.89], *t*(24) = −1.63, *p* = 0.116, two-tailed, *Hedges’ g* = −0.619. The Bayesian estimate of the true difference between state anxiety scores was 7.12 with a 95% credible interval (−16.14, 1.89) and the associated Bayes factor was 1.23, providing no evidence that the data were more probable under the null hypothesis (no difference in state anxiety score) than the alternative hypothesis.

To investigate whether any immediate anxiogenic effect was observed, differences in anxiety between baseline and day one was compared using an independent samples *t* test (Table 3). Group 1 (FP) (*n* = 12) showed a mean reduction in anxiety score of 7.65 compared to a reduction of 1.51 in group 2 (PF) (*n* = 13). The mean difference in anxiety between groups was not statistically significant, *M* = −6.13, [95% CI −17.23, 4.96], *t*(23) = −1.14, *p* = 0.265, two-tailed, *Hedges’ g* = −0.443. The Bayesian estimate of the true difference between state anxiety scores was 6.13 with a 95% credible interval (−17.23, 4.96) and the associated Bayes factor was 2.07, providing no evidence that the data were more probable under the null hypothesis (no difference in state anxiety score) than the alternative hypothesis.

### 3.4. Trait Axiety Levels from Baseline to 30 Days Post Infusion End

A paired samples *t* test of trait anxiety scores at 30 days post infusion end (*M* = 46.92, *SD* = 17.65, *n* = 21) was lower than baseline trait anxiety (*M* = 55.47, *SD* = 12.13) and was statistically significant, *M* = −8.55 [95% CI −3.41, −13.70], *t* = −3.47, *p* = 0.002, *Hedges’ g* = −0.756.

## 4. Discussion

Data indicate that in participants taking, on average, the same daily diazepam dose, flumazenil manages state anxiety symptoms within a clinically acceptable range during BZD withdrawal and significantly decreases trait anxiety following BZD withdrawal. The management of anxiety symptoms during and after BZD withdrawal is key to successful discontinuation as BZDs are often initially prescribed for the management of anxiety disorders [11,15]. Therefore, increases in anxiety during and after BZD withdrawal may lead to unsuccessful discontinuation or relapse. As anxiety is one of the most common symptoms occurring during BZD withdrawal [36], it is paramount to determine whether flumazenil produces an anxiogenic response, as worsening of anxiety may result in poorer outcomes.

Fortunately, unlike the anxiogenic response that has been shown with bolus injections of flumazenil in previous studies [18,19,20,21,22,23], there was no evidence that flumazenil had a clinically significant anxiogenic effect at low continuous doses and may actually result in anxiolysis. This is apparent when examining the 95% confidence interval for the difference between state anxiety in group 1 (FP) and group 2 (PF), which suggests that there is only a small chance of any clinically important increase in anxiety. It has been suggested that differences of 10 and above on the state anxiety scale are clinically significant [26,27]. The upper limit of the 95% confidence intervals were 1.89 and 4.96 and, therefore, not clinically significant.

Bolus doses of flumazenil in the range of 1–2 mg have been shown to occupy approximately 50% of BZD receptors [37,38]. This occupancy is what has been shown to induce panic, anxiety, and withdrawal reactions. While flumazenil is delivered in similar dose ranges in BZD withdrawal, but over several hours or continuously, low-dose continuous flumazenil infusion would not reach a receptor occupancy near 50%. This is due to a combination of a short half-life [39] and the slow infusion rate.

While state anxiety did not show any significant differences or changes between groups before the crossover period, trait anxiety was significantly reduced at one month. This could suggest that flumazenil has some anxiolytic action. The theory of the anxiolytic action of flumazenil has been described previously [34] and postulates that chronic stress produces changes in GABA_A_ receptor subtype expression leading to an altered response to endogenous substances. This results in a paradoxical anxiogenic effect of the endogenous neurosteroid, allopregnanolone, which usually acts as a positive allosteric modulator of the GABA_A_ receptor. This effect may be reversed by flumazenil via removal of altered GABA_A_ receptor subtypes [40]. The findings are consistent with this theory, which suggests that an anxiolytic action of flumazenil would last beyond the duration of the infusion, until chronic stress is experienced and altered GABA_A_ receptor subtypes repopulate [34]. As such, a reduction in trait anxiety beyond the flumazenil infusion may be due to the ability of allopregnanolone to modulate the GABA_A_ receptor in its typical anxiolytic manner. Alternatively, an anxiolytic action could also be attributed to a reversal of tolerance to BZDs, allowing for a greater anxiolytic efficacy when taken post-flumazenil infusion; however, the majority of participants were not using BZDs at one month [41] and tolerance to the anxiolytic effects of BZDs rarely occurs [6].

The anxiolytic action of flumazenil in BZD withdrawal has not been commonly researched. One case series investigated anxiety levels using the STAI–S and showed a downward trend in state anxiety during the flumazenil infusion [42]. Participants received a continuous flumazenil infusion (2 mg/24 h) delivered intravenously for up to 96 h. To our knowledge, the present study uses the highest dose of flumazenil (4 mg/24 h) for BZD withdrawal and is the first to explicitly measure anxiety during and after treatment in a randomised control setting. This study provides evidence that low-dose flumazenil does not induce an anxiogenic response in chronic BZD users that have significantly reduced their BZD use and are taking, on average, approximately 15 mg of diazepam per day. This is despite the dose being as much as four times greater (range: 1–4 mg/24 h) than that observed in similar studies of low-dose flumazenil for BZD withdrawal [25]. Therefore, it would be reasonable to assume that even lower doses delivered at the same rate are unlikely to be anxiogenic.

## 5. Limitations

It is difficult to assess the anxiolytic action of flumazenil during BZD withdrawal due to the inherent anxiogenic nature of the withdrawal syndrome and the confounding use of BZDs, which are anxiolytic. Additionally, the heterogeneity of benzodiazepines and z-drugs used by participants at baseline make it difficult to assess the specific substances that flumazenil should be used for with respect to withdrawal. Irrespective of this, anxiety levels did not significantly increase despite a significant reduction in BZD use in both groups. While the results did not detect any evidence for a clinically significant anxiogenic response to flumazenil, the possibility of an anxiolytic or anxiogenic action cannot be eliminated completely. This is likely due to the small sample size; however, it will serve well to inform on future sample sizes for adequately powered trails on the effects of low-dose flumazenil during BZD withdrawal. Trait anxiety levels 30 days post flumazenil infusion may be confounded by some participants still using BZDs; however, irrespective of BZD use after the infusions, trait anxiety levels were significantly decreased compared to when participants were using daily BZDs at baseline. Even if participants never ceased BZDs, a decrease in trait anxiety is still a noteworthy finding and suggests some anxiolytic effect.

## 6. Future Research

We found some evidence that flumazenil may have an anxiolytic effect based on a significant decrease in trait anxiety following the infusion. Future research should investigate whether an anxiolytic effect of flumazenil exists during the flumazenil infusion and confirm whether any clinically relevant anxiogenic effect exists. This could be expanded to investigate the effect of flumazenil on different benzodiazepine drugs and z-drugs individually. Given the lowest effect size from the state anxiety data analysis was moderate (0.443), future studies should recruit at least 81 participants in each group to maintain a power of 80%. The sample would need to be larger to further examine the action of flumazenil on multiple individual benzodiazepines and z-drugs. Furthermore, the anxiolytic effect occurring after 30 days post infusion end may warrant further investigation into the use of flumazenil as an anxiolytic for chronic BZD users.

## 7. Conclusions

There is a growing body of evidence for the use of continuous low-dose flumazenil infusions for BZD withdrawal and to our knowledge, this is the first study to explicitly explore anxiety during and after treatment. The data supports a conclusion that flumazenil does not significantly increase state anxiety to a clinically significant level and may even decrease state anxiety during BZD withdrawal, unlike bolus doses of flumazenil. Additionally, we found evidence that trait anxiety is decreased from baseline at approximately 30 days after flumazenil infusion, which suggests an anxiolytic effect. These findings need to be confirmed in larger randomised control trials.

## Figures and Tables

**Table 1:** Baseline demographic characteristics for the randomised groups receiving flumazenil followed by placebo (group 1) or placebo followed by flumazenil (group 2).

Variable	Group 1 (*n* = 13)	Group 2 (*n* = 13)
Female	8 (61.5)	9 (69.2)
Age (mean years, SD)	46.8 (12.5)	55.2 (11.2)
BMI ^$,%^ (mean, SD)	25.0 (6.0)	29.0 (10.3)
Diazepam daily equivalent dose (mean mg, SD) ’	51.4 (74.1)	33.0 (26.4)
Length of BZD use (mean years, SD) ^%^	4.4 (3.9)	9.3 (7.0)
Main BZD used		
- Alprazolam	0 (0)	1 (8)
- Clonazepam	5 (38)	8 (62)
- Diazepam	4 (31)	1 (8)
- Lorazepam	2 (15)	1 (8)
- Oxazepam	1 (8)	0 (0)
- Zolpidem ^§^	1 (8)	1 (8)
- Zopiclone ^§^	0 (0)	1 (8)
Diagnosed psychiatric condition	13 (100)	13 (100)
- Depression ’	8 (62)	7 (54)
- Anxiety ’	6 (46)	5 (38)
- Posttraumatic stress disorder ’	6 (46)	3 (23)
- Bipolar disorder ’	2 (15)	3 (23)
- Substance use disorder ’	3 (23)	0 (0)
- Other ’	4 (31)	0 (0)
Other daily substance use in the last week ^#^		
- Alcohol	0 (0)	3 (23)
- Marijuana	1 (8)	0 (0)
- Opioids (prescribed and/or illicit)	4 (31)	0 (0)

Note: Data were given as the count (percentage) unless otherwise indicated. ^$^ Calculated as weight in kilograms divided by height in metres squared. ^%^ Data missing for more than one participant. ^§^ Technically not classified as a BZD. ’ Data missing for one participant. ^#^ No self-reported daily drug use for amphetamines (including MDMA), cocaine, hallucinogens, and inhalants.

**Table 2:** Summary of diazepam use from baseline and mean diazepam use over the first eight-day infusion period.

	Baseline BZD Use (mg)	Mean BZD Use Over First Eight-Day Infusion (mg)	Difference in BZD Use between Baseline and First Eight-Day Infusion (mg)
**Group 1 (FP)**	51.4 (74.1, 12)	15.6 (12.5, 12) *	−35.8 (69.1, 12)
**Group 2 (PF)**	33.0 (26.4, 13)	15.7 (11.8, 13) **	−17.3 (20.2, 13)

Note: Results reported as mean (SD, *n*). * Denotes significantly different mean compared to baseline in the same group (*p* < 0.05). ** Denotes significantly different mean compared to baseline in same group (*p* < 0.005).

**Table 3:** Mean state anxiety scores at baseline and day 1 and mean state anxiety score over day 1, 4, and 8.

	Baseline	Day 1	Average Anxiety
**Group 1 (FP)**	57.5 (9.6, 13)	49.5 (10.5, 12)	50.1 (7.32, 13)
**Group 2 (PF)**	48.1 (14.4, 13)	46.5 (14.8, 13)	47.8 (11.18, 13)

Note: Results reported as mean (SD, *n*).

## Data Availability

Clinical trial registration number (anzctr.org.au): ACTRN12616001560482. Date registered: 11 November 2016.
